# Variations in wait times for imaging services: a register-based study of self-reported wait times for specific examinations in Norway

**DOI:** 10.1186/s12913-023-10284-2

**Published:** 2023-11-23

**Authors:** Bjørn Hofmann, Ingrid Øfsti Brandsaeter, Elin Kjelle

**Affiliations:** 1 Centre of Medical Ethics, Faculty of Medicine, University of Oslo, PO Box 1130, Oslo, N-0318 Norway; 2 Institute for the Health Sciences, Norwegian University of Science and Technology (NTNU), Gjøvik, Norway

**Keywords:** Wait times, MRI, CT, Ultrasound, Radiograph, Effective care, Safety, Quality

## Abstract

**Background:**

While the number of medical images has increased substantially, the demand has outpaced access, resulting in long wait times in many countries. Long wait times are a key problem for patient safety and quality of care as they can result in prolonged suffering, delayed diagnosis and treatment, as well as poorer prognosis and loss of lives. Surprisingly, little is known about wait times for imaging services.

**Objective:**

Investigate wait times for specific imaging services in Norway and to compare wait times with the total number of examinations and their development over time.

**Methods:**

Data from the wait time registry at the Norwegian Directorate of Health from 2018 to 2021 as well as data on outpatient imaging provided by the Norwegian Health Economics Administration (HELFO) and in-patient data afforded by fourteen hospital trusts and hospitals in Norway were analysed. Data include the total number of imaging examinations according to the Norwegian Classification of Radiological Procedures (NCRP). Analyses were performed with descriptive statistics.

**Results:**

Wait times vary through the months of the year. Conventional X-ray (XR) had the shortest wait times (3.0-4.4 weeks), and Magnetic Resonance Imaging (MRI) and ultrasound (US) had the lengthiest (8.7–12.0 and 7.9–11.4 weeks respectively). The wait times were lengthiest during the summer and winter holidays. Variations in wait times were also found for specific examination types between Norway’s four public health regions. In addition, there was variation over time within the health regions. The wait times with the private health providers were substantially lower than with the public health providers. From 2018 to 2021, the wait time for MRIs increased by 6.6%, while the number of examinations (per 10,000) increased by 8.6%. Those regions with the highest number of examinations per 1,000 inhabitants per year had the lowest wait times.

**Conclusion:**

Wait times for diagnostic imaging procedures varied with time, region, and modality in Norway from 2018 to 2021. Long wait times may entail many negative consequences for patients, professionals, and the healthcare system. Reducing long wait times is an obvious way to improve the quality, safety, and efficiency of care.

## Background

Medical imaging is an invaluable asset in modern patient care, as it can enable accurate diagnostics, expedite vital treatment, and reduce morbidity and mortality [1]. While the number of images has increased substantially worldwide [2], the demand has outpaced the access, resulting in long wait times in many countries [3–6].

Long wait times can result in prolonged suffering, delayed diagnosis and treatment, risk of incidental findings, and poorer prognosis and loss of lives, as well as lack of sustainability [7–9]. Moreover, wait times are central in patients’ complaints about radiological services [10, 11] while shorter wait times are associated with higher patient satisfaction [12].

In affluent parts of the world, long wait times partly result from extensive inappropriate imaging and low-value examinations [13]. This represents a profound challenge for patient safety and quality of care in modern healthcare systems [14]. Moreover, long wait times have negative effects, such as “queue jumping” [15], e.g., applying unfair methods to obtain quicker access to care.

Despite being a pronounced problem for patients, referrers and performers of medical imaging, few studies document wait times. Wait times for imaging have been included in reported general concerns for wait times for healthcare services [16]. Moreover, the general potential impacts of wait times for patient care have been highlighted [4] and the implications for the prognosis of specific diseases, such as lung cancer [7] and endometrioid endometrial cancers [9] have been documented as well as the difference in fair access to care [14, 17].

The objective of this study was to provide knowledge of wait times for imaging services in general by investigating the temporal and geographical variation of wait times for specific outpatient imaging services in Norway and to relate wait times with the total number of examinations. The research questions are as follows:

1. How do the wait times for various imaging modalities (conventional X-ray (XR), computed tomography (CT), Magnetic Resonance Imaging (MRI), and ultrasound (US)) vary over the months of the year?
2. What are the differences in wait times for specific examinations for different public health regions in Norway?
3. How do wait times vary over the years (2018–2021) for public and private providers?
4. How do wait times relate to the volume and population of the specific imaging examinations?

## Methods

### Setting

Norway has universal health coverage, and most health services are publicly funded through taxes [18] providing essential medical care to all citizens [19]. Specialist services are organized within the specialist care, governed by four regional hospital trusts who run 19 hospital trusts [20]. Each hospital trust has one or more radiological departments. Table 1 provides an overview of the number of modalities for public and private providers in Norway in July 2021. Imaging referrers in primary care, such as general practitioners (GP) and manual therapists, are organized under local municipalities.

**Table 1:** Number of MRI machines, CT scanners, and XR modalities for public and private imaging providers, as well as population 2021. Number of imaging machines is provided by the Norwegian Radiation and Nuclear Safety Authority (DSA). Population numbers are provided by Statistics Norway

Modality	MRI	CT	XR	Population
**Health region (public providers)**	92	90	147	5,390,620
Region North	16	18	20	481,764
Central Region	17	17	28	736,668
Region West	18	19	26	1,121,466
Region South-East	41	36	73	3,050,722
**Total Private Imaging Providers**	44	19	16	
**Other (ideal organizations)**	4	6	8	
**Total number**	140	115	171	

In 2021 there were 28 private image providers, that perform about 20–25% of all outpatient examinations [21]. A significant portion of these examinations is commissioned by public health services to ease the pressure on public providers [20]. Private imaging centres also offer imaging paid by private insurance or out-of-pocket, which allows shorter wait times for some patients. About 10% of the population have private health insurances [18].

### Data

Data on wait times is provided by the Norwegian Directorate of Health. Institutions providing imaging services in or for the public health service must report wait times and how long they are expected to be valid for. Wait time data are available for specific non-acute examinations: XR, US, CT of the head, chest, abdomen, pelvis, kidney and urinary tract, and MRI of the head, abdomen, spine, pelvis, extremities (arms and legs).

Wait time data are available in whole weeks for each radiology department or institution and are provided on request to the Norwegian Directorate of Health. All data are also publicly available at [22].

The data collected included number of examinations, Norwegian Classification of Radiological Procedures (NCRP) code (codes used to classify diagnostic examinations), modality, hospital/imaging centre, and whether they were in- or out-patient.

Data on out-patient imaging is provided by the Norwegian Health Economics Administration (HELFO). Examinations paid out-of-pocket or by private insurance are not included.

In-patient imaging data was collected from fourteen hospital trusts and hospitals in Norway covering 3,985,054 persons (73% of the population) and makes up 69% of the in-patient data set. Missing in-patient data was extrapolated based on collected out- patient data and population characteristics for each health trust.

### Analysis

Simple descriptive statistical analyses were performed in Microsoft Excel 2016 [23]. Averages are estimated based on reported data for each provider each month.

## Results

Each of the following sections addresses one of the research questions.

### Wait times variation over the months of the year

**Fig. 1:**
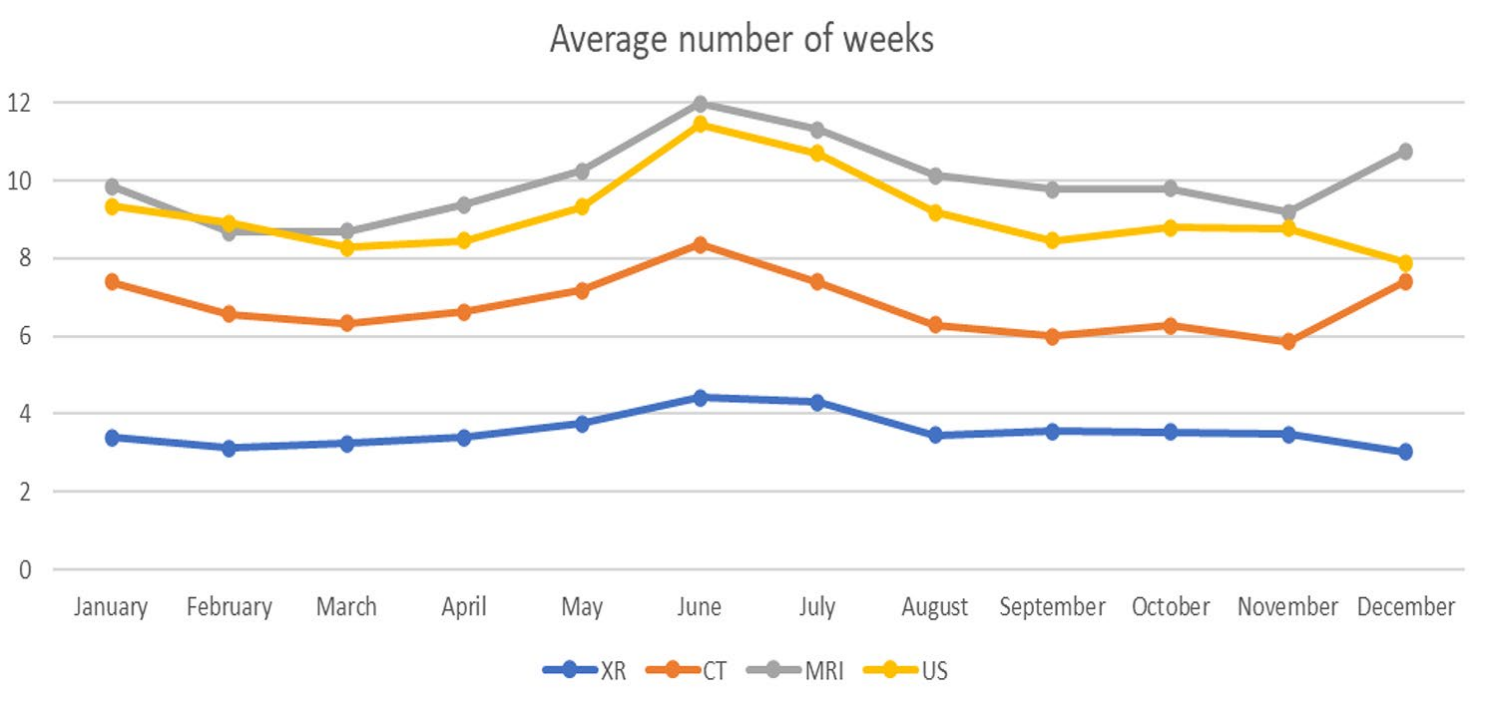
Average wait times in number of weeks throughout the months of the year for main modalities for specific examinations for 2021

Average wait times in number of weeks throughout the months of the year for main modalities XR, US, MRI (pelvis, abdomen, head, extremities, and spine), and CT (pelvis, Kidney/urinary tract, abdomen, head, and chest) for 2021 are shown in Fig. 1. XRs have the shortest wait times (3.0-4.4 weeks) and MRI and US the lengthiest, 8.7–12.0 weeks and 7.9–11.4 weeks respectively. That is, the wait times variation is 46% for XR, 38% for MRI, and 44% for US with longer wait times during the summer and winter holidays.

### Regional wait time variations

Average wait times for specific examinations for the various regions and for public and private imaging providers as well as total average wait times in number of weeks for four main modalities for 2021 are shown in Table 2. MRI have the lengthiest while XR has the shortest wait time in all health regions. Region South-east have the lengthiest wait time in all modalities except for US, where region West have the lengthiest wait time. The variation in wait times between specific CT and MRI examinations is very small within each region. In total, the standard deviations for XR, US, CT, and MRI are 0.42, 1.0, 0.74, and 1.0 respectively.

**Table 2:** Average wait times in number of weeks for specific examinations and for the four main modalities in the four public health regions in 2021 for public and private providers

Examination\Region	Region North	Central Region	Region West	Region South-East	Average (Public)	Average (Private)
CT pelvis	6.5	8.0	6.8	14.9	9.1	3.0
CT kidney/urinary tract	6.5	8.0	6.8	15.0	9.1	3.1
CT abdomen	6.5	8.0	6.9	14.7	9.0	3.2
CT head	6.1	7.3	6.8	14.3	8.6	2.9
CT chest	6.6	8.0	6.7	14.8	9.0	3.0
MRI pelvis	11.2	12.0	15.1	20.3	14.7	5.3
MRI abdomen	11.1	12.0	15.1	20.2	14.6	5.4
MRI head	11.1	12.2	14.0	20.2	14.4	5.4
MRI extremities	11.3	12.1	13.7	20.3	14.4	5.3
MRI spine	11.2	11.9	14.0	20.2	14.3	5.3
Total average XR	4.5	3.1	3.1	6.7	4.4	1.9
Total average US	5.2	7.0	12.7	10.9	9.0	6.5
Total average CT	6.4	7.9	6.8	14.7	9.0	3.0
Total average MRI	11.2	12.0	14.4	20.2	14.5	5.3

### Variation in wait times for MRIs by public health providers and private providers

The wait times varied somewhat over the years from 2018 to 2021 as shown in Table 3. Longer wait times in 2020 are due to restrictions during the SARS-COV-2 pandemic. Wait times altered little in Region North during the main year of the pandemic, while they increased substantially in Region South-East while the number of MRIs per 10,000 was only moderately reduced. On average the number of MRIs per 10,000 inhabitants were reduced with 5.6% from 2019 to 2020 while they were reduced with 7.2% in Region South-East. The wait time in Region South-East increased with 150.5% and the average wait time for the public and private imaging providers increased on average 100.7% and 24.5% from 2019 to 2020. Wait times are in general substantially lower at the private compared to the public health providers.

**Table 3:** Average wait times in number of weeks for the four public health regions and average public and private wait times for registered MRI examinations from 2018 to 2021

	2018	2019	2020	2021
**Public Imaging Providers**	13.6	14.3	28.7	14.5
Region North	9.5	10.3	10.4	11.2
Central Region	7.8	8.2	10.9	12.0
Region West	12.6	11.9	14	14.4
Region South-East	19.8	18.8	47.1	20.2
**Private Imaging Providers**	4.3	4.9	6.1	5.3
**Total average wait time**	7.3	7.4	12.4	9.9

### Imaging use, population, and wait times for MRI examinations

The number of examinations increased in all regions from 2018 to 2021: 12% in Region North, 16.4% in Central Region, 2.7% in Region West, and 4.7% in Region South-East.

**Fig. 2:**
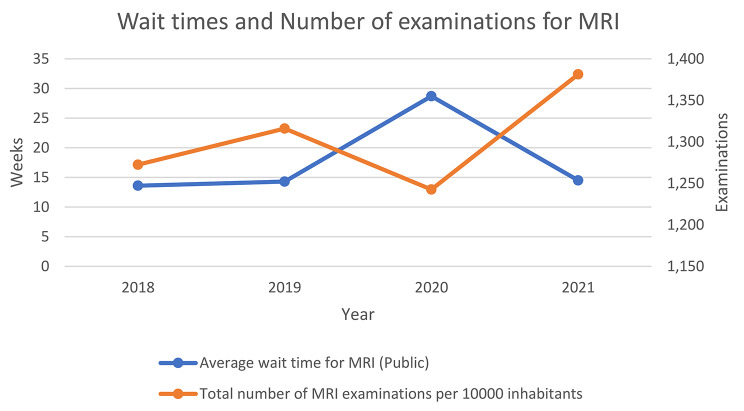
Wait times in weeks and number of MRI examinations per 10,000 inhabitants for the public providers from 2018 to 2021

With exception from the pandemic year 2020, wait times for MRI increased with 6.6% while number of examinations per 10,000 increased with 8.6% from 2018 to 2021 in the public health services. Wait times increased with 23.2% in the private providers from 2018 to 2021, but wait times were still about one third for the private providers compared to the public providers. Fig. 2 shows the wait times related to the number of examinations per 10,000 inhabitants from 2018 to 2021. It shows a clear effect of the covid-19 pandemic in 2020.

The number of MRIs vary greatly over the four regions as do the sizes of the populations and wait times. Table 4 provides an overview over the number of MRIs, number of inhabitants, and MRIs per inhabitant in the four regions. MRIs vary from 178,090 in Region South-East to 38,484 in Region North in 2021 while the population is 3,050,722 and 481,764. The number of MRIs per inhabitant per year is highest in the Central Region with an average of 0.09 per person per year in 2021 while Region West and South-East both had 0.06.

**Table 4:** Overview of total number of MRIs, inhabitants, MRIs per inhabitant, and wait times for MRIs for the various public health regions for 2021

Total regional wait times for MRIs	Total number of public outpatient MRIs 2021	Total number of inhabitants in region	Average number of MRIs per 1000 inhabitants	Average wait times (weeks)
Region North	38 484	481 764	80	11.2
Central Region	66 044	736 668	90	12.0
Region West	62 462	1 121 466	56	14.4
Region South-East	178 090	3 050 722	58	20.2

**Fig. 3:**
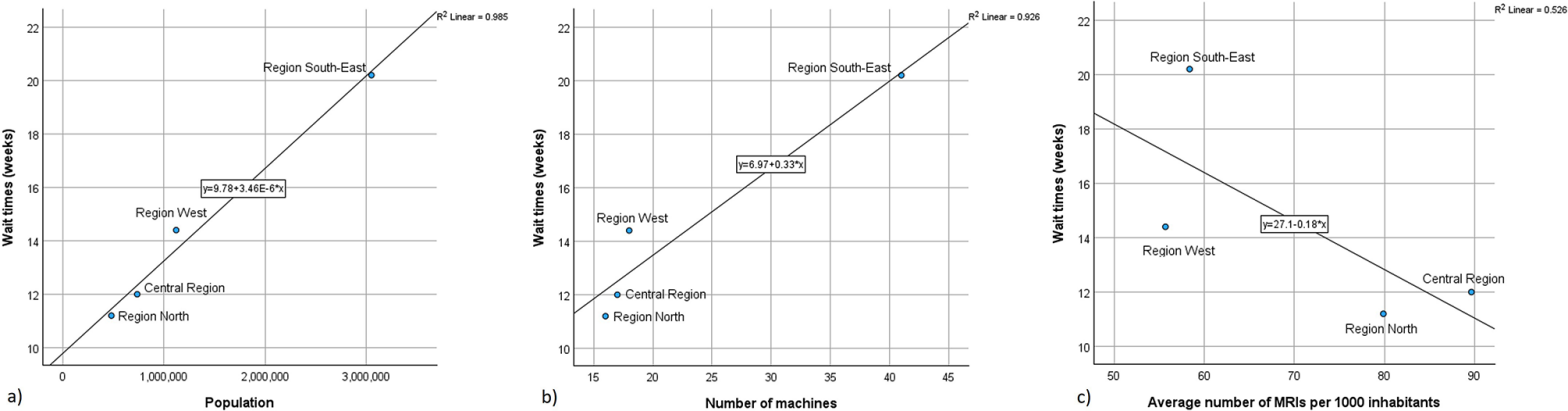
Scatter plots for wait time (weeks) compared to a) population per region, b) number of MRI machines per region, and c) number of MRIs per 1000 inhabitantsfor 2021

The wait times in the public imaging providers appear to increase with number of MRI machines and with the number of inhabitants but decrease with number of MRIs per 1000 inhabitants in each region. Fig. 3 provides basic scatter plots for wait time (weeks) compared to population per region, number of MRI machines per region, and number of MRIs per 1000 inhabitants for 2021. Figure 3c indicates that Region South-East and West have higher wait times and lower use rates compared to Region North and Central Region.

While these results should be interpreted with care, the data indicates that there is a negative relationship between utilization and wait time, i.e., that those regions that have the lengthiest wait times have the lowest number of examinations per 1000 inhabitants per year and vice versa.

## Discussion

This study shows that wait times vary over the months of the year with 46% for XR,, 42% for CT, 38% for MRI, and 44% for US with longer wait times during the summer holidays. Differences across the four public health regions in Norway are substantial for the specific examinations and wait times vary over the years, from on average 7.3 weeks in 2018 to 9.9 weeks in 2021. Wait times are substantially lower for the private than the public health providers, and those regions with the highest number of examinations per 1,000 inhabitants per year had the lowest wait times (in 2021).

Wait times are very context sensitive, i.e., to the national and local health service organization. Hence, international comparison can be difficult. However, in general the results are in line with other studies of wait times [4, 6, 14, 16, 17, 24–26]. Norway is close to average amongst the waiting times for CT scans according to European Health Consumer Index 2018 [27].

The variation over the year shown in this study could be contingent on staffing as there are longer wait times during vacation periods [28], and similar effect on wait times throughout the year have been seen in the NHS [29]. 2020 was an exceptional year due to the pandemic and the authorities introduced measures to reduce contamination that affected wait times both in Norway [30] and internationally [31, 32]. However, it is interesting to note that wait times increased substantially while the total number of examinations did not change correspondingly (Table 3). The Number of examinations was reduced over three months during the spring of 2020 but rapidly went back to normal [33]. This indicates that there was a reasonable capacity at the imaging providers. Moreover, the regional health trusts with the lengthiest wait times also had the lowest number of examinations per 1000 inhabitants, which indicates some differences in efficiency. The reason might be that units with more specialized imaging procedures may have longer examination times per patient, due to more imaging sequences.

The reason why wait times are substantially lower with the private than with the public health providers is partly due to a division of tasks. The public health providers handle acute patients, in-patients, and more complex issues than the private health providers, who in general handle more ordinary and less severe or complex cases [20]. It is also important to underscore that the reported wait times are for non-acute cases and for out-patient services.

As displayed in Fig. 3 regions with high population and high equipment density have long wait times. However, regions with a high use rate tend to have lower wait times while regions having long wait times have lower use rates. According to the logical framework of Nuti et al. [24] this raises concern about efficiency and potential overactivity and should be investigated further.

Long wait times are a key problem for the health services as they undermine patient safety and quality of care, resulting in prolonged suffering, emotional stress, delayed diagnosis and treatment, as well as potentially poorer prognosis and loss of lives [7–9]. A study by Liddy et al. investigated the patients with chronic pain perspective on wait times and found that most patients had experienced an impact on day-to-day life and work or school. More than half worried about having a serious undiagnosed disease [26]. At the same time, we know that one source of long wait times is extensive inappropriate imaging or imaging of low value for the patient [13, 34]. This results in a double negative paradox: people who do not need examinations delay or block access for those who do, challenging patient safety and quality of care [14].

Waiting times in complex systems such as imaging services very much depend on the balance between the capacity and demand [35]. In general, capacity and demand can vary substantially between regions, making the application of use rates challenging. However, it is important to notice that Norway has a healthcare system with equity as a core value and a homogenous healthcare system and population [19].

Several measures to reduce wait times are available, such as wait time screening tools [36] and decision-support systems for addressing wait times [37]. Reducing unnecessary imaging such as inappropriate imaging or imaging with low value for the patient’s care can be one way to reduce long wait times. Earlier research has shown that 84 different imaging procedures can be of low value for specific patient groups, many with proportions of more than 50% having no effect on the patients’ further treatment or care [13, 38]. Moreover, a wide range of measures to reduce low value imaging, and thereby to free resources for high-value imaging, and reducing wait times, have been documented [39].

Limitations.

This study only includes wait times for a specific number of examinations. Wait times for other examinations are unknown. This is a general problem in other countries as well [40]. More reliable wait time data is necessary to assess and improve the quality and safety of imaging services. Moreover, wait time information is not easily accessible for the general population [41].

Another major limitation with this study is that wait times are not measured but reported by the care providers themselves. While there is a chance that providers would embellish reports, this would not benefit them when patients discover the discrepancy between reported and real wait times.

Moreover, wait times are not reported on exact the same date each month, and some providers do not report every month. However, while missing data occur (e.g., wait times for one month) they do not influence the average estimates including only reported data. Moreover, data include the covid-19 pandemic (2020) which undermines a wide range of statistical analysis. For more advanced study more accurate data are necessary.

The scatter plots in Fig. 3 should be interpreted cautiously as they give broad confidence intervals. However, they are consistent with the results for other years and modalities. While they of course do not indicate any general relationship between wait times, imaging equipment, population, and productivity, they report the imaging practice in Norway.

We have not defined what is meant by “long wait times” as this can vary for patients’ conditions (severity), disease progression, healthcare setting etc. This study has only provided an overview of the phenomenon “wait times,” and future and more detailed studies should investigate what qualifies as a “long” wait time in context.

Nonetheless, this study contributes to the scarce literature on wait times with new information about an issue which is of great challenge to the quality and safety of care.

## Conclusion

Wait times for diagnostic imaging vary throughout the year for all modalities, with XR having the shortest wait time, and MRI, CT, and US having the lengthiest wait time. In addition, wait times vary within and between the four public health regions in Norway. As this study indicates that long wait times are related to large populations and much diagnostic equipment, but low utilization rates, more research in wait times is warranted.

Long wait times can have negative consequences for the patient’s quality of life, diagnosis, and treatment. As low-value imaging is one reason for long wait times, measures to reduce these examinations is one way to shorten wait times.

## Data Availability

All data are publicly available from https://www.helsenorge.no/velg-behandlingssted/ventetider/.

## List of abbreviations

CT: Computed Tomography
GP: General Practitioners
MRI: Magnetic Resonance Imaging
US: Ultrasound
XR: Conventional x-ray
